# Gender‑Affirming Care as a Predictor of HIV Pre‑Exposure Prophylaxis Use and Adherence Among Young Trans Feminine Adults: A Coincidence Analysis

**DOI:** 10.1007/s11121-025-01814-x

**Published:** 2025-06-06

**Authors:** Alithia Zamantakis, Richard Do, Reiping Huang, Artur A. F. L. N. Queiroz, Brian Mustanski

**Affiliations:** 1 Department of Medical Social Sciences, Institute for Sexual and Gender Minority Health and Wellbeing, Northwestern University, 625 N. Michigan Ave., Ste. 1400, Chicago, IL 60615, USA; 2 Department of Medical Social Sciences, Northwestern University, Chicago, IL, USA; 3 Northwestern Quality Improvement, Research, and Education in Surgery (NQUIRES), Feinberg School of Medicine, Northwestern University, Chicago, IL, USA; 4 American College of Surgeons, Chicago, IL, USA; 5 Center of Population Sciences for Health Equity, College of Nursing, Florida State University, Tallahassee, FL, USA; 6 Department of Psychiatry and Behavioral Sciences, Northwestern University, Chicago, IL, USA

**Keywords:** Transfeminine persons, HIV, Pre-exposure prophylaxis, Hormone replacement therapy, Gender-affirming surgery, Puberty blockers

## Abstract

We used coincidence analysis to explore whether various forms of gender-affirming care (GAC) in the presence or absence of medical mistrust facilitate HIV pre-exposure prophylaxis (PrEP) use and adherence. Using secondary data collected between 2014 and 2024 from the RADAR Cohort Study, we performed two crisp-set coincidence analyses with 86 trans feminine young adults for PrEP use and 24 trans feminine young adults for PrEP adherence. Our final model for PrEP use explained over 90% of participants who had used PrEP in the past 6 months with 60% consistency. This model identified receipt of hormone replacement therapy (HRT) OR being on parental insurance as predictors of PrEP use. We identified two final models for PrEP adherence, which explained 50% of participants with 83% consistency: (1) past receipt of puberty blockers OR high suspicion of medical providers in the absence of parental insurance; (2) current or past receipt of HRT in the absence of barriers to GAC and the absence of parental insurance. Our study highlights the significant role of GAC in facilitating PrEP use and adherence among trans feminine individuals. Specifically, HRT and the absence of parental insurance emerged as key predictors, underscoring the need for integrated and accessible GAC to enhance PrEP uptake and adherence in this population.

## Introduction

Transgender women (TW) experience large disparities in HIV prevalence, prevention, and treatment (Baral et al., 2013; Becasen et al., 2019; Centers for Disease Control & Prevention, 2021). The first ever National HIV Behavioral Surveillance System’s transgender cycle found 42.2% of TW surveyed across seven major US cities to be living with HIV, compared to an HIV prevalence of 0.4% nationally (Centers for Disease Control & Prevention, 2021, 2024). Efforts to target disparities in HIV prevention include the dissemination of pre-exposure prophylaxis (PrEP), a once-daily pill or bimonthly injection, that can reduce chances of HIV acquisition by 99% if taken as prescribed (Landovitz et al., 2021; Mayer et al., 2020). However, just 32% of TW have been found to use PrEP despite 92% of HIV-negative TW being aware of PrEP (Centers for Disease Control & Prevention, 2021). Less is known about HIV prevalence among nonbinary individuals; however, some research has found nonbinary youth to use PrEP at lower rates than TW (Aryal et al., 2023; Fitch et al., 2022).

Disparities in PrEP use and adherence exist for several reasons, including concerns regarding PrEP interaction with hormone replacement therapy (HRT; Zamantakis et al., 2023), daily stressors (Colson et al., 2020), adverse PrEP side effects (Nieto et al., 2020; Ogunbajo et al., 2021; Wood et al., 2019), and stigma (Jalil et al., 2022; Quinn et al., 2023; Watson et al., 2023). PrEP marketing campaigns have emphasized its usage among men who have sex with men (MSM), contributing to notions that PrEP is only used for MSM (Watson et al., 2023; Zamantakis et al., 2023).

Research has found that marginalized populations, broadly, experience additional extrinsic stressors (i.e., daily necessities, poverty, housing, employment), which retain priority over affording regular doses of PrEP, and intrinsic stressors (i.e., mental health conditions, depression, anxiety, post-traumatic stress disorder), which inhibit motivation to adhere to PrEP (Colson et al., 2020; Ogunbajo et al., 2021; Zamantakis et al., 2023). Minority stress theory may help explain the mechanism by which racial, gender, and sexual orientation discrimination lowers PrEP adherence motivation (Hunter et al., 2021; Meyer, 1995).

Recent research has shown, though, that gender-affirming care (GAC) may increase PrEP use and adherence (Connolly et al., 2020; Sevelius, Glidden, et al., 2021b; Starbuck et al., 2022; Zamudio-Haas et al., 2023). Unfortunately, the literature on mechanisms in which GAC mediates PrEP use and adherence is ambiguous. Research has suggested that TW who utilize GAC were more likely to engage in PrEP-based discussions with their healthcare providers, therefore increasing trust in healthcare systems and improving engagement in HIV prevention (Poteat & Radix, 2020; Sevelius, Keatley, et al., 2016a, 2016b). In addition, by bundling PrEP with HRT within a single clinic, uptake and adherence among TW may be improved by simplifying medication regimens and providing accurate information on drug interactions (Sevelius, Deutsch, et al., 2016a; Zamudio-Haas et al., 2023).

We used coincidence analysis (CNA) to further explore whether various forms of GAC facilitate PrEP use and adherence. In comparison to traditional statistical analyses (e.g., regression analytic methods), CNA uses Boolean logic (Baumgartner & Falk, 2023). CNA analyzes which variables (referred to as factors or conditions) in conjunction (i.e., together) or in disjunction (i.e., separately) predict an outcome. While traditional statistical analyses can detail the odds of an outcome occurring or associations between variables, CNA takes an entire set of factors into account and identifies pathways in the forms of factor conjunctions that lead to an outcome (referred to as solutions; Whitaker et al., 2020). In identifying pathways, CNA examines which conditions are *necessary* and *sufficient* to produce an outcome (Mackie, 1965; Whitaker et al., 2020). CNA is prime for intersectional research. As CNA identifies differential pathways to an outcome, it enables an analysis in which multiple pathways may produce the same outcome for different sub-samples (Quintana, 2023). In other words, it is possible that what works for Black transgender women may be different from what works for nonbinary Latine individuals.

We performed a crisp-set (i.e., using binary factors with values 0 and 1) CNA to explore whether various forms of GAC in the presence or absence of medical mistrust and healthcare utilization predict PrEP use and/or adherence.

## Methods

### Participants

Data for these analyses were taken from RADAR, an ongoing longitudinal cohort study of young sexual and gender minorities assigned male at birth in Chicago, including young sexually minoritized men, transgender women, and nonbinary AMAB youth, that began in 2014 (Mustanski et al., 2019). The primary objective of the cohort study is to apply a multilevel perspective to a syndemic of health issues associated with HIV and substance use. Study visits occur every 6 months until a participant turned 30 years of age after which they switched to an annual visit schedule. Institutional Review Board (IRB) approval was received for this secondary data analysis from the Northwestern University IRB in April 2024.

For this study’s purposes, we only included data from participants who identified as a gender other than male/man and who responded to items assessing GAC access and barriers (*n* = 156). After removing participants missing any response to items measuring medical mistrust, the final sample for the PrEP use CNA included 86 participants. The number of participants removed was due to variations in which respondents were asked questions regarding medical mistrust depending on when they were enrolled in RADAR.

We also conducted a PrEP adherence CNA, which only included data from participants who reported PrEP use in the past 6 months. After removing participants who did not report currently using PrEP, the final sample for the PrEP adherence CNA included 24 participants.

### Outcomes

PrEP use was operationalized as whether the participant has taken any type of PrEP in the past 6 months. Participants were able to respond “yes” or “no.” For PrEP adherence, participants were asked, “When was the list time you missed a dose of your PrEP medication?” Participants could respond (1) within the past week, (2) within the last 4 weeks, (3) greater than 4 weeks, (4) over 3 months ago, or (5) never. PrEP adherence was dichotomized as “0” for those who missed a dose either within the past week or the past 4 weeks and “1” for those who missed a dose either never or more than 4 weeks ago. We dichotomized PrEP adherence in this way to distinguish between those who had missed a dose during their most recent prescription (i.e., the past 4 weeks) and those who had not. While this loses some granularity, it also maintained a sufficient distribution of the data to allow for analysis. The full data dictionary is available in Table 1.

### Factors

Factors included lifetime HRT use, source of HRT (e.g., GAC setting, general medical setting), whether they have had any gender-affirming surgery, silicone injections, or injectable fillers for gender-affirming purposes (henceforth, “gender-affirming operations”), lifetime use of puberty blockers, and whether they have experienced any barriers to accessing gender-affirming care (e.g., financial barriers, lack of insurance coverage). Lifetime HRT use, whether they have had any gender-affirming operations, and lifetime use of puberty blockers were dichotomized as “yes” or “no.” Source of HRT included the following responses: “gender-affirming clinic,” “a general doctor’s office or clinic,” and/or “a friend or non-medical setting.” Source of HRT was calibrated as a multivalue factor to account for each response item, as well as a fourth, “multiple sources.” Finally, participants were asked to respond to a list of barriers they had experienced in accessing gender-affirming operations, including “insurance did not cover this treatment,” “high financial cost of treatment,” or “concerns about acceptance from family and/or friends.” Due to limited variability within the data, we summed the number of barriers marked by respondents and dichotomized at the mean (mean: 4; IQR: 1, 8).

We included two sub-measures to account for negative perceptions of PrEP (i.e., PrEP stigma) and positive perceptions of PrEP, dichotomized at the mean due to a lack of an empirically recommended cutoff and to preserve balance in data variability. For negative perceptions of PrEP, the mean equaled 2.08 (IQR: 1.29, 2.61). For positive perceptions of PrEP, the mean equaled 4.1 (IQR: 3.67, 4.67).

We included three sub-measures of medical mistrust, including “suspicion of medical providers,” “lack of support from providers,” and “group disparities in care.” These sub-measures are part of a larger Group-Based Medical Mistrust measure (Thompson et al., 2004) and are further described in Table 1. We dichotomized each of these at the mean due to a lack of an empirically recommended threshold for this measure and to preserve balance in data variability. For “suspicion of medical providers,” the mean equaled 13 (IQR: 6, 18). For “lack of support from providers,” the mean equaled 8 (IQR: 6, 9). For “group disparities in care,” the mean equaled 11 (IQR: 9, 13.75).

Finally, we included three items to account for healthcare utilization, including whether the participant currently has a primary care provider (PCP), whether they disclose their gender identity to their PCP, and whether the participant is covered by their parent’s insurance. Each of these were dichotomized based on the response items of “yes” and “no.”

### Demographics

Participants were able to report gender as woman, trans woman, man, nonbinary, gender-nonconforming, and/or another gender not listed. Race was reported by participants as Black/African American, white, Asian American/Pacific Islander, Alaskan Native/Indigenous/Native American, and/or Hispanic/Latinx. Educational attainment was reported as less than a high school diploma, high school diploma or GED, some trade school or college, or more. Age was reported by participants in addition to year of birth. Sexual orientation was reported as gay, lesbian, heterosexual/straight, queer, bisexual, pansexual, asexual, and/or another sexual orientation not listed.

### Data Analysis

We conducted a crisp-set CNA in R software using CNA package 3.5.3.4 (Ambuehl and Baumgartner, 2018; 2024; R Core Team, 2021). Based on the calibration (i.e., “operationalization”) described above for each factor, data were transformed in R into binary and multivalue factors. Our complete R script is available in Supplementary Appendix SA1. We used the minimally sufficient condition or “msc” approach to determine which factors to include in the final analysis based on coverage and consistency (Miech, 2023).

Consistency refers to how reliably a solution or model predicts an outcome. Coverage refers to the number of cases with the outcome that fit the solution or model (Whitaker et al., 2020). For PrEP use, we included factors that “rose to the top” (defined as appearing frequently above a coverage of 0.03; range of 0 to 0.09). For PrEP adherence, this was defined as appearing frequently above a coverage of 0.2 (range of 0 to 0.4). We selected factors based on coverage of mscs, as all solutions produced in the initial msc had consistencies of 1.0.

Multivalue factors like source of HRT were binarized based on the msc. Just as with crisp-set (i.e., binary) factors, we examined which factors “rose to the top” based on coverage. The only two conditions (i.e., values of the factor) that rose to the top for source of HRT were receipt of HRT from a general doctor’s office or clinic and receipt of HRT from multiple settings. The other sources of HRT (receipt from a GAC clinic or from a friend/non-medical setting) did not appear. As such, we binarized source of HRT as “0” = receipt of HRT from a friend, non-medical setting, or GAC clinic and “1” = receipt of HRT from a general doctor’s office, clinic, or multiple settings.

A total of 10 factors were included for PrEP use and 7 for PrEP adherence (see Table 1 for a full list in the data dictionary, as well as for justification of how factors were calibrated; see Supplementary Table S1 and Supplementary Table S2 for data input for analysis).

We began with a dual consistency-coverage threshold of 95%. We then reduced coverage, followed by consistency, by 5% each until solutions were identified. For PrEP adherence, we identified three solutions with consistency above 63.6% and coverage above 62.5%. Due to imbalance in the PrEP use outcome (i.e., only 25.6% of cases had a positive outcome), we performed CNA with prevalence-adjusted consistency and coverage thresholds (De Souter & Baumgartner, 2024). This allowed us to identify three models with consistency above 60% and coverage above 55%. We aimed to identify solutions with consistency and coverage above 70% but were unable to do so for PrEP use due to data imbalances. The results presented are from the CNA performed with prevalence-adjusted consistency and coverage thresholds. The script for the weighted consistency and coverage is included in Supplementary Appendix SA1.

The final models presented are those that we selected based on a balance of coverage, consistency, and complexity. Complexity refers to the number of conditions present in a model. To prevent overfitting, we did not select models that had a high complexity (i.e., 9 or higher). After limiting complexity below this, we identified models that had high(er) consistency without forgoing coverage (i.e., we did not select models that had high consistency but only covered one case in which the outcome was present).

### Sensitivity Tests

We performed substitution sensitivity tests for the three medical mistrust sub-measures, recalibrating them to one above the mean, as these three measures did not have empirical or theoretical basis for originally defining at the mean (Huang, 2023). This sensitivity test allows us to determine if a change in the operationalization (i.e., calibration) of the sub-measures results in a difference of results, such that the models may be subject to change due to “fuzziness” in operationalization. We only did so for PrEP adherence, due to one of these measures appearing in the final model.

### Positionality Statement

The authorship team of this manuscript comprises a diverse group. Alithia Zamantakis is a transgender woman from a multiracial white and Latinx family. Richard Do is a cisgender Vietnamese man completing his high school education from Evergreen Valley High School. Reiping Huang is a cisgender woman. Artur Queiroz is a queer immigrant scholar and an HIV-rights activist. He is a PrEP user for almost a decade. Brian Mustanski is a white, cisgender sexual minority man.

## Results

### Demographics and Participant Characteristics

#### PrEP Use.

Most participants were 25 years old or older (54.7%; range = 18–31), identified as gender non-conforming, nonbinary, or another gender (e.g., agender; 58.5%), and had completed at least some college or trade school, if not more (59.3%). The largest proportion of participants were non-Hispanic Black/African-American (*n* = 37; 43.0%). Only 11 participants had undergone some form of gender-affirming operation, including electrolysis, silicone injection use, fillers, and other injections for gender-affirming purposes. See Table 2 for complete demographics.

#### PrEP Adherence.

Most participants were 24 years old or younger (58.4%; range = 18–31), identified as either women and/or TW (66.7%), and had had completed at least some college or trade school, if not more (62.5%). The largest proportion of participants were non-Hispanic white (*n* = 9; 37.5%). Only three participants had undergone some form of gender-affirming operation, but most had currently or previously received HRT (*n* = 15; 62.5%). A quarter had previously received puberty blockers.

### PrEP Use Models

A total of 86 participants were included in the PrEP use analysis. Just under a quarter (24.4%) reported PrEP use in the past 6 months. Our final model (model 1 in Table 3) explained over 90% of participants who had used PrEP in the past 6 months (19/21, coverage = 90%), with 60% consistency. This solution included lifetime HRT use OR being one one’s parents’ insurance. Table 3 details two additional models that consisted of one condition each.

Participants for whom parental insurance predicted PrEP use were primarily 25 years of age or older with a high school diploma, GED, or less education. Those participants for whom lifetime HRT use predicted PrEP use were primarily 24 years of age or younger and had completed at least some trade school or college, if not more. Misfires (i.e., those who were covered by their parents’ insurance or were currently or previously receiving HRT but who were not currently using PrEP) were primarily 24 years of age or younger, Black TW who had completed at least some trade school or college, if not more.

### PrEP Adherence Models

A total of 24 participants were included in the PrEP adherence analysis. Forty-one percent were categorized as PrEP adherent (10/24). Our two final models (models 1 and 2 in Table 4) explained 50% of participants who adhered to PrEP (5/10, with 50% coverage), with 83% consistency. Model 1 included current or past use of puberty blockers OR being on one’s parents’ insurance AND the participant reporting greater suspicion of providers. Model 2 included lifetime HRT use AND lower barriers to GAC AND being on one’s parents’ insurance.

Regarding model 1, those for whom receipt of puberty blockers predicted adherence were primarily Black, gay/lesbian TW over 24 years of age with no higher education. Those for whom not being on their parent insurance and having higher suspicion of providers predicted adherence were primarily gay/lesbian TW.

Regarding model 2, those for whom receipt of HRT and lower barriers to GAC and who were not on their parent’s insurance predicted adherence were primarily TW of color, over 24 years of age with no higher education.

### PrEP Adherence Sensitivity Test

After rerunning the CNA with the newly calibrated medical mistrust measures, suspicion of providers no longer appeared in the top models. Model 1 also no longer appeared as a solution, due to this. These results detail that suspicion of providers, and potentially the other medical mistrust measures, are sensitive to definition changes.

## Discussion

Significantly, our study corroborated prior research identifying HRT use as a predictor of PrEP use and adherence (Connolly et al., 2020; Sevelius, Glidden, et al., 2021b; Starbuck et al., 2022; Zamudio-Haas et al., 2023). This highlights the need for future research to attend to the implementation of integrated HRT and PrEP care. The goals of the National Ending the HIV Epidemic Initiative include the need to scale up PrEP use and adherence and to mitigate disparities across minoritized populations (The White House, 2022). Achieving this will require implementation strategies, or methods to enhance the reach, adoption, implementation, and maintenance of evidence-based interventions like integrated HRT and PrEP (Fauci, 2013; Glasgow et al., 1999; Proctor et al., 2013; Wood et al., 2020).

We additionally found that HRT use in the presence of no barriers to GAC and not being on one’s parents’ insurance predicts PrEP adherence. This conjunction highlights the need to reduce barriers to GAC broadly while integrating PrEP and HRT, specifically. Furthermore, we identified puberty blockers as a predictor of PrEP adherence. Use of puberty blockers requires familial support to access care as a minor. It may be that familial support is the mechanism through which past puberty blocker use influences PrEP adherence. Further qualitative research is needed in this regard.

Of note, increased suspicion of providers in the absence of parental insurance also predicted PrEP adherence. While it may seem counterintuitive that being suspicious of providers would facilitate PrEP adherence, which requires engagement with providers, it is possible that being aware of medical injustice allows patients to better prepare to cope with the impacts of it. Additional research may be needed to understand if there is a difference between being on insurance broadly and being on parental insurance. Being on parental insurance may impact PrEP adherence by continuing to provide access to care for those under 24. Prior research, though, has found that familial support may also play an important role in PrEP adherence (Wood et al., 2020).

Overall, our analyses detail a need for HIV prevention providers to better integrate GAC and PrEP to increase PrEP use and adherence. One way to do so may be with telemedicine to facilitate access to GAC and PrEP, which may enable nonbinary individuals to access specialized GAC and PrEP without worrying about hostile or unsupportive environments (Inwards-Breland et al., 2024). Furthermore, providers may need to identify strategies to increase familial support of young trans feminine adults in their pursuit of HIV prevention interventions. Taken as a whole, this work details the need for providers to take a holistic approach to HIV prevention.

### Limitations

Our findings should be considered with the following limitations. First, while we included gender-affirming operations as a factor, the data were so imbalanced that the absence of gender-affirming operations as a predictor of PrEP use or adherence may be due to limitations in the distribution of the data. Only 11 participants for PrEP use and just 2 for PrEP adherence had undergone some form of gender-affirming operation. This is likely due to the younger age of the participants. CNA operates best when there is greater balance in the distribution of an outcome (generally recommended to be around a 60/40% split). When the distribution is skewed, it makes it more difficult for the algorithm to identify factors that predict an outcome.

Second, the low consistency for the PrEP use solutions signifies that there may be factors that were not included in the model that need to be considered as potential predictors. It also signifies that the data imbalances may result in the CNA being unable to find a complete solution. Low consistency highlights that a solution is less reliable at predicting an outcome. Lower consistency can occur due to imbalances in data, non-inclusion of causal factors, or inclusion of spurious factors. Despite this, the results do highlight HRT use, puberty blockers, parental insurance, and suspicion of providers as difference-makers for PrEP use and adherence.

### Generalizability of the Models

Whitaker et al. (2020) note that “familiarity with cases helps to evaluate generalizability—by, for example, justifying that cases included in the analysis are homogenous with respect to potential confounders—and to interpret solutions generated by mathematical modeling.” One of the strengths of this manuscript is our analysis of the intersectional, demographic differences across cases within and between models. For the PrEP use and PrEP adherence models, our participants were homogenous vis-a-vis age (i.e., all young adults, aged 18–31) and gender (i.e., all transgender and nonbinary individuals assigned male at birth). There was greater variation in education and race/ethnicity.

Regarding the PrEP use model, we examined not only which participants were covered by certain pathways within the model, but we also identified that misfires were primarily Black TW. It is unclear why this is the case, but further qualitative research would aid in understanding the reason for this. Regarding the PrEP adherence models, the only commonality among those cases that adhered to PrEP but were not explained by either model 1 or 2 was that they were 24 years old or younger. It is thus possible that our models are only generalizable to young adults over 24 years of age and that other factors not included in these models may explain PrEP adherence for younger individuals. The next step in our study is to carry out qualitative interviews with those who were either not covered by the selected models or who were misfires within both the PrEP use and adherence analyses to better understand what additional factors may explain this.

## Conclusion

Our study concluded that HRT use and puberty blockers in the presence or absence of parental insurance and medical mistrust are predictors of PrEP use and adherence, emphasizing the need for integrated care. Additionally, reducing barriers to gender-affirming care (GAC) and ensuring individuals are not reliant on parental insurance are crucial for improving PrEP adherence. At a time in which GAC for youth and young people has become illegal under a recent executive order titled, “Protecting Children from Chemical and Surgical Mutilation,” these results are even more critical (The White House, 2025). Building on prior research that has detailed the importance of GAC to transgender and nonbinary populations’ mental health (Green et al., 2022; Tordoff et al., 2022), antiretroviral treatment (ART) adherence and viral suppression (Maiorana et al., 2021; Sevelius et al., 2014, 2022, 2021a), and overall wellbeing (De Vries et al., 2014), we detail the importance of GAC to increasing PrEP use and adherence. GAC facilitates better health outcomes and behaviors for transgender and nonbinary individuals. Reduced access to GAC will only limit the possible interventions and implementation strategies that could be tested and deployed to further transgender and nonbinary health and wellbeing.

## Supplementary Material

Appendix SA1

## Figures and Tables

**Table 1:** Data dictionary

Variable type	Name	Value	Definition	Justification	Sensitivity	Included in PrEP use CNA?	Included in PrEP adherence CNA?
Factor	HRT use	1 = No 2 = Yes (either past or current)	If participant has ever taken hormone replacement therapy	Combined three versions of No, because whether they want to in the future or not does not really matter as to whether they’ve used it or not		X	X
Factor	Location of hormone receipt	0 = Does not use HRT, GAC setting, or non-medical settings 1 = General medical setting or multiple settings	Location from which participant has received their HRT	Calibrated based on multivalue msc		X	
Factor	Prior surgery or injection for GAC	0 = No surgery or injection 1 = Has had surgery or injections	If the participant has ever had top surgery, bottom surgery, silicone injections, other types of injections, or other surgical modifications. Other surgical modifications include nose (*n* = 2), hips (*n* = 1), butt (*n* = 4), castration/orchiectomy (*n* = 3), facial feminization surgery (*n* = 13), vocal surgery (*n* = 2), electrolysis/laser (*n* = 4), forehead reduction (*n* = 1)	Too few of participants who have had surgery or injections, so we combined them into a single factor		X	
Factor	Puberty blocker use	0 = No or not in age range 1 = Yes (either past or current)	If the participant has ever had puberty blockers as a youth/adolescent	Combined three versions of No, because whether they want to in the future or not does not really matter as to whether they’ve used it or not. Combined those outside the age range for puberty blockers with “no,” because there’s not a meaningful difference between the no’s and “not able to because it doesn’t apply to my age”		X	X
Factor	PrEP stigma	0 = 1–2 1 = > 2	Score of 7 items assessing participant perception of PrEP. The higher the score, the greater the stigma	Calibrated at median			
Factor	Positive attitudes toward PrEP	0 = 2.333 to 4 1 = > 4	Score of 3 items assessing participant perception of PrEP. The higher the score, the greater the positive attitude toward PrEP in comparison to stigma	Calibrated at median			
Factor	Patient currently has PCP	0 = No 1 = Yes	Does the participant currently have a primary care provider?	No change from raw data		X	X
Factor	Barriers to care	0 = < 2 1 = > 2	Does the participant have any barrier to any type of GAC?	Calculated the number of total barriers across all barriers that patients experienced and then used the mean as a threshold	Changed to mean (4)		
Factor	Patient out to provider	0 = No and I don’t know 1 = Yes and somewhat	Is the participant out to their PCP as an LGBTQ person?	No and I don’t know are essentially the same for a quantitative purpose. Combined somewhat and yes because breaking them up resulted in too fragmented of a factor		X	X
Factor	Patient on parent’s insurance	0 = No 1 = Yes	Is the participant covered by their parent or guardian’s healthcare insurance?	No change from raw data		X	
Factor	Group-based medical mistrust,”suspicion”	0 = 6–12 1 = > 13	Participant responses to the following: People of my ethnic group shouldn’t confide in doctors and healthcare workers because it will be used against them. People of my ethnic group should be suspicious of information from doctors and health care workers. People of my ethnic group cannot trust doctors and health care workers. People of my ethnic group cannot trust doctors and health care workers. People of my ethnic group cannot trust doctors and health care workers. People of my ethnic group cannot trust doctors and health care workers. Responses are summed 6–30. The higher the sum, the more suspicion they have of health care providers	Calibrated at the mean because there’s no empirical or theoretical number by which to otherwise base it	Substitution: Changed to 1 above mean	X	X
Factor	Lack of support from providers	0 = 3–7 1 = > 8	Participant responses to the following: Doctors and health care workers sometimes hide information from patients who belong to my ethnic group. Doctors have the best interests of people of my ethnic group in mind (reverse coded). I have personally been treated poorly or unfairly by doctors or health care workers because of my ethnicity. Responses are summed 3–15. The higher the score, the greater the perceived lack of support from providers	Calibrated at the mean because there’s no empirical or theoretical number by which to otherwise base it	Substitution: Changed to 1 above mean	X	
Factor	Group disparities in care	0 = 3–10 1 = > 11	Participant responses to the following: People of my ethnic group receive the same medical care from doctors and health care workers as people from other groups (reverse coded). People of my ethnic group are treated the same as people of other groups by doctors and health care workers (reverse coded). In most hospitals, people of different ethnic groups receive the same kind of care (reverse coded). Responses are summed 3–15. The higher the score the greater perceived group disparities in care	Calibrated at the mean because there’s no empirical or theoretical number by which to otherwise base it	Substitution: Changed to 1 above mean	X	X
Outcome	PrEP use	0 = No 1 = Yes	Has the participant taken any type of PrEP in the past 6 months?	No change from raw data		X	
Outcome	PrEP adherence	0 = within past week to 4 weeks ago 1 = Greater than 4 weeks to over 3 months ago or never	When was the last time the participant missed a dose of their PrEP medications? If they took only a portion of a dose on one or more of these days, that is counted as missing a dose	Data were not granular enough to calculate how many doses missed, so calibrated based on whether a dose has been missed since last prescription (i.e., within last 4 weeks)			

**Table 2:** Participant demographics and characteristics

	PrEP use *n* (%)	PrEP adherence *n* (%)
Total *N*	86	24
Age		
25 years old +	47 (54.7)	10 (41.6)
24 years old and younger	39 (45.3)	14 (58.4)
Gender identity		
Woman/transgender woman	35 (40.5)	16 (66.7)
Gender nonconforming, genderqueer, nonbinary, or another gender	51 (58.5)	8 (33.3)
Sexual orientation		
Straight/heterosexual	23 (26.7)	0 (0.0)
Bisexual, pansexual, and/or queer	37 (43.0)	9 (37.5)
Gay/lesbian	21 (24.4)	15 (62.5)
Questioning or another sexual orientation	5 (5.8)	0 (0.0)
Educational attainment		
Some college, trade school, or more	51 (59.3)	15 (62.5)
GED, high school diploma, or less	35 (40.7)	9 (37.5)
Race/ethnicity		
Non-Hispanic Black/African American	37 (43.0)	8 (33.3)
Non-Hispanic white	25 (29.1)	9 (37.5)
Hispanic/Latine	13 (15.1)	4 (16.7)
Non-Hispanic Other	7 (8.1)	3 (12.5)
Gender-affirming operation		
Yes	11 (12.7)	3 (12.5)
No	75 (77.3)	9 (77.5)
HRT		
Yes	43 (50.0)	15 (62.5)
No	43 (50.0)	9 (37.5)
Puberty blockers		
Yes	22 (25.6)	6 (25.0)
No	64 (74.4)	18 (75)
Location from which HRT was received^a^		
General doctor’s office (e.g., primary care)	9 (10.5)	1 (4.2)
Friend or other non-medical setting	1 (1.2)	0 (0.0)
Gender-affirming clinic	0 (0.0)	0 (0.0)
Multiple settings	4 (4.7)	1 (4.2)

a Only 14 participants responded to this question for PrEP use and 2 for PreP adherence

**Table 3:** Use models

Case	Age^a^	Gender^b^	Education^c^	Sexuality^d^	Race/ethnicity^e^	M1: Parent insurance	M2: HRT use	Model 1: M1 + M2^h^	Use
25	1	1	2	2	1	0	1	1	1
11	1	1	2	3	3	1	1	1	1
13	1	2	2	3	1	0	0	0	1
1	1	1	1	2	3	0	1	1	1
26	1	1	1	2	3	0	1	1	1
2	0	1	2	2	4	0	1	1	1
3	1	1	2	2	2	0	1	1	1
27	0	1	1	1	3	0	1	1	1
16	1	1	1	3	3	0	1	1	1
5	1	1	1	3	3	0	0	0	1
28	0	2	2	2	2	1	0	1	1
29	0	2	2	2	2	1	0	1	1
6	1	1	1	3	3	0	1	1	1
7	0	2	2	2	2	1	0	1	1
8	0	1	1	3	2	0	1	1	1
17	1	1	1	3	2	0	1	1	1
18	0	1	2	2	2	1	1	1	1
20	0	2	2	2	2	1	0	1	1
21	0	2	2	1	4	1	0	1	1
30	0	1	1	2	4	0	1	1	1
31	0	2	2	2	3	1	0	1	1
32	1	1	2	3	4	0	0	0	0
33	1	1	2	3	3	0	0	0	0
34	1	1	2	2	1	0	1	1	0
35	1	1	2	4	3	0	0	0	0
36	1	1	1	1	3	0	1	1	0
37	1	1	1	1	3	0	1	1	0
38	1	1	1	1	3	0	0	0	0
39	0	1	1	3	3	0	1	1	0
40	1	2	2	4	4	0	0	0	0
41	0	2	2	2	4	0	0	0	0
42	1	1	1	3	4	0	1	1	0
43	1	1	2	2	3	0	1	1	0
44	0	2	1	2	4	0	0	0	0
45	0	2	2	2	4	1	0	1	0
46	0	1	1	2	2	0	1	1	0
47	0	2	2	2	4	0	0	0	0
48	0	1	1	1	3	1	1	1	0
49	1	1	1	3	3	0	0	0	0
50	1	1	1	3	3	0	1	1	0
51	1	1	2	2	3	0	0	0	0
52	0	2	2	2	2	1	1	1	0
53	1	2	2	2	2	1	0	1	0
54	0	2	2	2	2	1	0	1	0
55	1	1	2	2	1	0	1	1	0
56	0	1	2	2	2	1	1	1	0
57	0	1	1	1	3	0	1	1	0
58	1	2	1	1	3	0	0	0	0
59	0	2	2	2	2	0	0	0	0
60	0	1	2	1	3	1	1	1	0
61	1	1	2	1	1	0	1	1	0
62	0	1	2	3	4	0	1	1	0
63	1	1	1	3	3	0	1	1	0
64	0	1	2	3	3	0	1	1	0
65	0	1	2	3	3	1	1	1	0
66	0	1	1	3	3	0	1	1	0
67	0	2	2	1	2	1	0	1	0
68	0	1	1	1	3	0	0	0	0
69	1	2	2	2	4	0	0	0	0
70	0	2	2	1	2	0	0	0	0
71	1	2	1	1	3	1	1	1	0
72	0	1	1	2	3	0	1	1	0
73	1	2	2	2	2	0	0	0	0
74	1	1	2	3	1	0	1	1	0
75	1	2	1	2	3	0	0	0	0
76	0	1	1	3	3	0	1	1	0
77	0	1	2	1	3	1	0	1	0
78	0	2	2	1	4	0	0	0	0
79	0	2	2	2	2	1	0	1	0
80	1	1	2	3	4	0	1	1	0
81	0	2	2	2	2	0	0	0	0
82	0	2	1	4	4	0	0	0	0
83	0	1	2	2	4	1	1	1	0
84	0	2	1	2	1	0	0	0	0
85	1	1	1	1	3	0	1	1	0
86	1	1	2	1	3	0	1	1	0
87	0	1	2	3	2	0	0	0	0
88	0	1	2	4	4	0	0	0	0
89	1	1	2	3	3	0	1	1	0
90	1	1	1	3	3	0	1	1	0
91	1	2	2	2	2	0	0	0	0
92	0	2	2	1	2	1	0	1	0
93	0	2	2	2	2	0	0	0	0
94	0	2	1	1	2	1	0	1	0
95	0	2	1	1	2	1	0	1	0
96	0	2	1	2	3	0	0	0	0

* Using Boolean operators, CNA identifies whether x OR y, or perhaps x AND y, lead to the selected outcome. The + is used to represent or. The represents and. In this analysis, we utilize crisp set (i.e., binary 0 and 1) and multivalue factors based on the characteristics of the raw data. M represents minimally sufficient conditions for the outcome. S represents solutions produced from the coincidence analysis

a Age: 0 represents those 24 and younger; 1 represents those 25 and older

b Gender: 1 represents women/trans women; 2 represents gender non-conforming, genderqueer, and nonbinary individuals

c Education: 1 represents GED/diploma or less; 2 represents trade school, some college, or higher

d Sexuality: 1 represents gay/lesbian; 2 represents bisexual, pansexual, and/or queer

e Race/ethnicity: 1 represents Native American/Asian American/Multiracial; 2 represents white; 3 represents Black/African American; 4 represents Latina/x

f Consistency: 60.7%; coverage: 38%

g Consistency: 64.4%; coverage: 55%

h Consistency: 60%; coverage: 90%

**Table 4:** Adherence models

Case	Age^a^	Gender^b^	Education^c^	Sexuality^d^	Race/ethnicity^e^	M1: Blockers	M2: Parent insurance^*^ suspicion	M3: HRT use^*^ barriers^*^ parent insurance	Model 1: M1 + M2^f^	Model 2: M3^g^	Adherence
1	1	1	1	1	3	1	1	1	1	1	1
2	0	1	2	1	4	0	1	1	1	1	1
3	1	1	2	1	2	0	0	0	0	0	1
4	0	2	2	1	1	0	0	0	0	0	1
5	1	1	1	2	3	1	0	1	1	1	1
6	1	1	1	2	3	1	0	1	1	1	1
7	0	2	2	1	2	0	0	0	0	0	1
8	1	1	1	2	2	0	0	1	0	1	1
9	0	2	2	1	3	0	0	0	0	0	1
10	0	1	1	1	3	1	0	0	1	0	1
11	1	1	2	2	3	0	0	0	0	0	0
12	0	1	1	2	3	0	0	1	0	1	0
13	1	2	2	2	1	0	0	0	0	0	0
14	1	1	2	2	4	1	0	0	1	0	0
15	0	2	2	1	2	0	0	0	0	0	0
16	1	1	1	2	3	0	0	0	0	0	0
17	0	1	1	2	2	0	0	0	0	0	0
18	0	2	2	1	1	0	0	0	0	0	0
19	0	1	2	1	2	0	0	0	0	0	0
20	0	2	2	1	2	0	0	0	0	0	0
21	0	2	2	1	4	0	0	0	0	0	0
22	0	1	1	1	4	1	1	0	1	0	0
23	1	1	2	1	2	0	0	0	0	0	0
24	0	1	2	1	2	0	0	0	0	0	0

* Using Boolean operators, CNA identifies whether x OR y, or perhaps x AND y, lead to the selected outcome. The + is used to represent or. The represents and. In this analysis, we utilize crisp set (i.e., binary 0 and 1) and multivalue factors based on the characteristics of the raw data. M represents minimally sufficient conditions for the outcome. S represents solutions produced from the coincidence analysis

a Age: 0 represents those 24 and younger; 1 represents those 25 and older

b Gender: 1 represents women/trans women; 2 represents gender non-conforming, genderqueer, and nonbinary individuals

c Education: 1 represents GED/diploma or less; 2 represents trade school, some college, or higher

d Sexuality: 1 represents gay/lesbian; 2 represents bisexual, pansexual, and/or queer

e Race/ethnicity: 1 represents Native American/Asian American/Multiracial; 2 represents white; 3 represents Black/African American; 4 represents Latina/x

f Consistency: 83%; coverage: 50%
