# Supplementary material for: Gender‑Affirming Care as a Predictor of HIV Pre‑Exposure Prophylaxis Use and Adherence Among Young Trans Feminine Adults: A Coincidence Analysis
Source: Prev Sci. Author manuscript; Available in PMC 2025 Jul 15. (PMC12245996; doi:10.1007/s11121-025-01814-x)
Supplement: Appendix SA1 [file NIHMS2088508-supplement-Appendix_SA1.docx]

Supplemental Appendix SA1

R Script

#### Calibration ####

# clean environment

rm(list =ls())

cat("\014")

# set working directory

getwd()

dt1 <- GAC4PrEP_Input_USE

dt1 <- GAC4PrEP_Input_ADHERE

dt1 <- GAC4PrEP_sensitivity_USE

dt1 <- GAC4PrEP_sensitivity_ADHERE

#### PrEP use msc 6/2024 ####

names(dt1)

dim(dt1)

msc<-msc(mvcna(dt1,ordering=list("USE"),

strict=TRUE, suff.only=TRUE, maxstep=c(3,4,10)))

### rearrange

msc<-arrange(msc, desc(outcome), complexity, desc(coverage), desc(consistency))

#---- export list

wb <- createWorkbook()

addWorksheet(wb, "msc_prep_use")

writeData(wb, "msc_prep_use", msc)

saveWorkbook(wb, "msc_prep_use.xlsx", overwrite = TRUE)

## stopped here to see which rise to the top

#### PrEP adherence msc ####

names(dt2)

dim(dt2)

msc<-msc(mvcna(dt2,ordering=list("ADHERE"),

strict=TRUE, suff.only=TRUE, maxstep=c(3,4,10)))

#---- export list

wb <- createWorkbook()

addWorksheet(wb, "msc_prep_adhere")

writeData(wb, "msc_prep_adhere", msc)

saveWorkbook(wb, "msc_prep_adhere.xlsx", overwrite = TRUE)

## stopped here to see which rise to the top

#### PrEP Adherence CNA ####

dim(dt1)

names(dt1)

## run CNA

cna(dt1, outcome ="ADHERE")

cna(dt1, con = .95, cov = .95, outcome = "ADHERE")

cna(dt1, con = .95, cov = .9, outcome = "ADHERE")

cna(dt1, con = .9, cov = .9, outcome = "ADHERE")

cna(dt1, con = .9, cov = .85, outcome = "ADHERE")

cna(dt1, con = .85, cov = .85, outcome = "ADHERE")

cna(dt1, con = .85, cov = .8, outcome = "ADHERE")

cna(dt1, con = .8, cov = .8, outcome = "ADHERE")

cna(dt1, con = .75, cov = .75, outcome = "ADHERE")

cna(dt1, con = .5, cov = .5, outcome = "ADHERE")

x <- rean_cna(dt1, outcome="ADHERE",

strict=T,attempt = seq(.9, .7, -0.1), output="asf")

M <- do.call(rbind, x)

##fr score

s1<-frscore(M$condition, dat=dt1)

s1

#------- combine outputs from cna, frscore

y <- condTbl(s1$models$model, dt1)

# round frscore to 3 decimals

s1$models$norm.score <- round(s1$models$norm.score, digit=3)

s1$models$con <- round(y$consistency, digit=3)

s1$models$cov <- round(y$coverage, digit=3)

s1$models$complex <- round(y$complexity, digit=3)

s1$models$fit2<-round(y$consistency*y$coverage, digit=3)

# add spaces around '+'

s1$models$model<-gsub("+", " + ", s1$models$model, fixed=TRUE)

#---- export list

wb <- createWorkbook()

addWorksheet(wb, "asf_9Xs")

writeData(wb, "asf_9Xs", as.data.frame(s1$models))

saveWorkbook(wb, "out_ADHERE.xlsx", overwrite = TRUE)

#### Weighted PrEP use CNA ####

dim(dt1)

names(dt1)

##remove and reinstall cna

remove.packages("cna")

install.packages("cna_3.5.3.4.tar.gz", repos = NULL, type ="source")

library(cna)

stopifnot(packageVersion("cna") == "3.5.3.4")

##define model building measures of weighted consistency and weighted c-coverage

def_w_measures <- ccDef_ratio(list(

cbind(c(1, 0, 0, 0, 0, 0),

c(1, 0, -99-1i, 0, -99-1i, 0)),

cbind(c(0, 1, 0, 0, 0, 0),

c(0, 1, 0, -99, -99, 0))))

##run cna

cna(dt1, con = 0.8, cov = 0.8, outcome = "USE", ccDef = def_w_measures)

cna(dt1, con = 0.8, cov = 0.75, outcome = "USE", ccDef = def_w_measures)

cna(dt1, con = 0.75, cov = 0.75, outcome = "USE", ccDef = def_w_measures)

cna(dt1, con = 0.75, cov = 0.7, outcome = "USE", ccDef = def_w_measures)

x <- rean_cna(dt1, outcome="USE",

strict=FALSE, attempt = seq(0.75, 0.45, -0.1), output="asf",

ccDef = def_w_measures)

#rbind combines rows cbind combines columns

M <- do.call(rbind, x)

table(M$outcome)

##19,647 asfs

##fr score, up to 100 asfs

s1<-frscore(M$condition, dat=dt1, maxsols =100)

#combine outputs from cna, frscore

y <- condTbl(s1$models$model, dt1, ccDef = def_w_measures)

# round frscore to 3 decimals

s1$models$norm.score <- round(s1$models$norm.score, digit=3)

s1$models$con <- round(y$consistency, digit=3)

s1$models$cov <- round(y$coverage, digit=3)

s1$models$complex <- round(y$complexity, digit=3)

s1$models$fit2<-round(y$consistency*y$coverage, digit=3)

# add spaces around '+'

s1$models$model<-gsub("+", " + ", s1$models$model, fixed=TRUE)

#---- export list

wb <- createWorkbook()

addWorksheet(wb, "asf_9Xs")

writeData(wb, "asf_9Xs", as.data.frame(s1$models))

saveWorkbook(wb, "out_USE.xlsx", overwrite = TRUE)
